# Biotransformation of (1*S*)-2-Carene and (1*S*)-3-Carene by *Picea abies* Suspension Culture

**DOI:** 10.3390/molecules161210541

**Published:** 2011-12-19

**Authors:** Marcela Dvorakova, Irena Valterova, David Saman, Tomas Vanek

**Affiliations:** 1 Laboratory of Plant Biotechnologies, Joint Laboratory of Institute of Experimental Botany, Academy of Sciences of the Czech Republic, v.v.i., and Research Institute of Crop Production, v.v.i., Rozvojova 263, 165 02 Prague 6, Czech Republic; Email: dvorakova@ueb.cas.cz (M.D.); 2 Department of Organic and Nuclear Chemistry, Faculty of Science, Charles University in Prague, Albertov 6, 128 43 Prague 2, Czech Republic; 3 Institute of Organic Chemistry and Biochemistry, Academy of Sciences of the Czech Republic, v.v.i., Flemingovo nam. 2, 166 10 Prague 6, Czech Republic; Email: david.saman@uochb.cas.cz (D.S.)

**Keywords:** biotransformation, carene, oxidation, *Picea abies*

## Abstract

Biotransformation of (1*S*)-2-carene and (1*S*)-3-carene by *Picea abies* suspension culture led to the formation of oxygenated products. (1*S*)-2-Carene was transformed slowly and the final product was identified as (1*S*)-2-caren-4-one. On the other hand, the transformation of (1*S*)-3-carene was rapid and finally led to the formation of (1*S*)-3-caren-5-one and (1*S*)-2-caren-4-one as equally abundant major products. The time-course of the reaction indicates that some products abundant at the beginning of the reaction (e.g. (1*S*,3*S*,4*R*)-3,4-epoxycarane and (1*R*)-*p*-mentha-1(7),2-dien-8-ol) were consumed by a subsequent transformations. Thus, a precise selection of the biotransformation time may be used for a production of specific compounds.

## 1. Introduction

Biotransformations are environmentally friendly methods to obtain valuable chemicals which are often used as flavours, fragrances and pharmaceuticals. There has been an extensive interest in biotransformations for they are chemo-, regio- and stereospecific and are a precious source of “natural products”, chemical compounds synthesized enzymatically without the use of toxic organic reagents and solvents [1].

Their significance is also inflicted in their variability. Biotransformations may be carried out by a vast range of organisms and on an ample variety of compounds, even exogenous ones [2,3]. Microbial systems may seem advantageous over the others because their biomass doubling times are short and methods for their genetic manipulation are well established [2]. On the other hand, plants possess unique enzymes which enhance their potential. For example, plants are involved in the biosynthesis of some very complicated pharmaceuticals such as paclitaxel or artemisinin [4,5].

(1*S*)-2-Carene (**1**) and (1*S*)-3-carene (**2**) are monoterpenes produced by conifers as components of their resin, which is engaged in plant defence against herbivores. On the other hand, oxygenated monoterpenes are the main source of aromas in spices and herbs, and they are in great demand for their antibacterial, antifungal and anticancer effects. The biotransformation of carenes by plant cell cultures may lead to oxygenated products which may themselves possess such properties or may be employed as the structural scaffolds in the synthesis of active compounds (for example: sesquiterpenoids, diterpenoids, β-lactam antibiotics) [6].

Since there exist only two reports on carenes transformation by plants [7,8], we have utilized *Picea abies* suspension culture to explore this area. The pine tree, *P. abies*, is the natural source of carenes and thus its enzymatic system is accustomed to their bioconversion and its cells are resistant to their toxicity to a great extent. Similar approach was taken by Miyazawa and Kano [9], who studied the biotransformation of (1*S*)-3-carene by larvae of *Spodoptera litura* which feeds on plants producing terpenes. Our study focuses on the identification of the biotransformation products and the determination of the dependence of the relative quantitative product yields on incubation time. The absolute configuration of the biotransformation products was assigned according to the synthesized reference compounds. For those compounds not synthesized, the configuration was assigned only when indisputable. The quantitative yields of (1*S*)-3-carene (**2**) biotransformation products are also given.

## 2. Results and Discussion

### 2.1. Autooxidation of Carenes

In case of (1*S*)-3-carene (**2**), the incubation with the medium without *P. abies* cells did not lead to any product formation. With (1*S*)-2-carene (**1**), a considerable amount (28%, relative) of (1*S*,4*R*)-2-caren-4-ol (**3**) was formed, but the rest of the starting material remained unchanged and no other products were produced.

### 2.2. Biotransformation of 2-Carene

**Figure 1:**
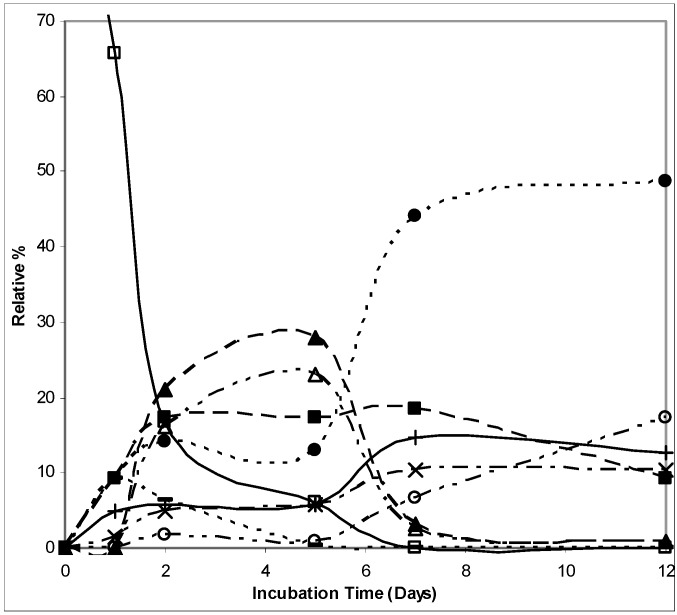
The relative abundance (in %) of (1*S*)-2-carene and its biotransformation products in time-course; Legend: □, (1*S*)-2-carene; ■, (1*R*)-*p*-menth-1(7),2-dien-8-ol; –, (1*S*,4*R*)-2-caren-4-ol; ×, *p*-cymen-8-ol; ○, (1*S*)-2-caranone; ●, (1*S*)-2-caren-4-one; +, (1*S*)-3-caren-2-one; ∆, (1*R*)-*p*-menth-2-en-7,8-diol; ▲, (1*R*,4*R*)-*p*-menth-2-en-1,8-diol.

The biotransformation of (1*S*)-2-carene (**1**) occurred rather slowly, although after two days, the majority (80% relative) of the starting material was consumed and transformed into oxygenated products. The relative quantitative yields of particular products differed with the biotransformation time-course. First, hydroxylated products such as (1*S*,4*R*)-2-caren-4-ol (**3**) and (1*R*)-*p*-mentha-1(7),2-dien-8-ol (**4**) were formed, which then underwent further transformations leading to (1*S*)-2-caren-4-one (**5**) as the major product (Figure 1).

**Scheme 1:**
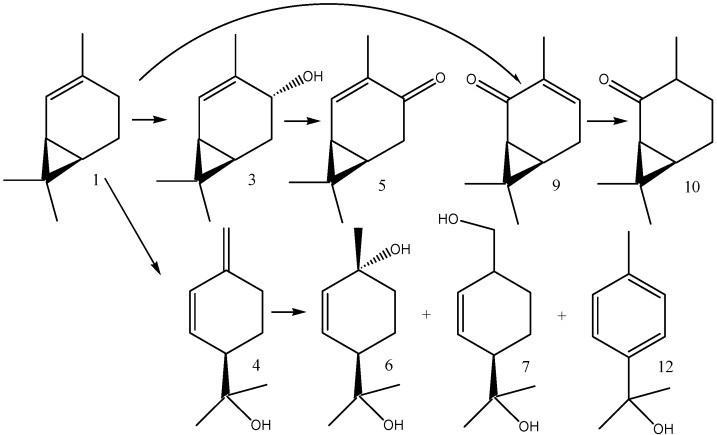
(1*S*)-2-Carene (**1**) and its biotransformation products—(1*S*,4*R*)-2-caren-4-ol (**3**), (1*S*)-2-caren-4-one (**5**), (1*S*)-3-caren-2-one (**9**), (1*S*)-2-caranone (**10**), (1*R*)-*p*-mentha-1(7),2-dien-8-ol (**4**), (1*R*,4*R*)-*p*-menth-2-en-1,8-diol (**6**), (1*R*)-*p*-menth-2-en-7,8-diol (**7**), and *p*-cymen-8-ol (**12**).

Dihydroxylated products, (1*R*,4*R*)-*p*-menth-2-en-1,8-diol (**6**) and (1*R*)-*p*-menth-2-en-7,8-diol (**7**), were generated within the first five days when their relative abundance reached 23 and 28%, respectively. Scheme 1 shows the structures of the biotransformation products of (1*S*)-2-carene (**1**), along with their formation relations.

2-Carene (**1**) was previously subjected only to a biotransformation by other plant tissue cultures: *Myrtillocactus geometrizans* and *Nicotiana tabacum* [7]. *Nicotiana tabacum* produced 2-caren-4-ol (**3**) and 3-caren-2-ol (**8**) as the major products while *Myrtillocactus geometrizans* gave predominantlytheir corresponding ketones. Our products of (1*S*)-2-carene (**1**) biotransformation were quite different. For example, we observed the formation of (1*S*)-2-caren-4-ol (**3**) in the medium itself, without the presence of *P. abies* cells. Thus, although this compound was also identified among the biotransformation products, it is uncertain whether it is caused by the simple presence of culture cells. Even though its abundance was the highest (10%), but not prominent, after the first day, and further decreased with time. Among our products were, after five days, substantial amounts of two diols, (1*R*,4*R*)-*p*-menth-2-en-1,8-diol (**6**) and (1*R*)-*p*-menth-2-en-7,8-diol (**7**). These were not formed by the other plant tissue cultures. At the end of the biotransformation time-course, the ketones became predominant. We also detected the formation of (1*S*)-2-caren-4-one (**5**) and (1*S*)-3-caren-2-one (**9**) like the aforementioned authors, however, the abundance of (1*S*)-3-caren-2-one (**9**) was in our case almost four times lower than that of (1*S*)-2-caren-4-one (**5**), which reached 48%. Also the abundance of (1*S*)-2-caranone (**10**, 17%) was higher than that of (1*S*)-3-caren-2-one (13%).

### 2.3. Biotransformation of 3-Carene

**Figure 2:**
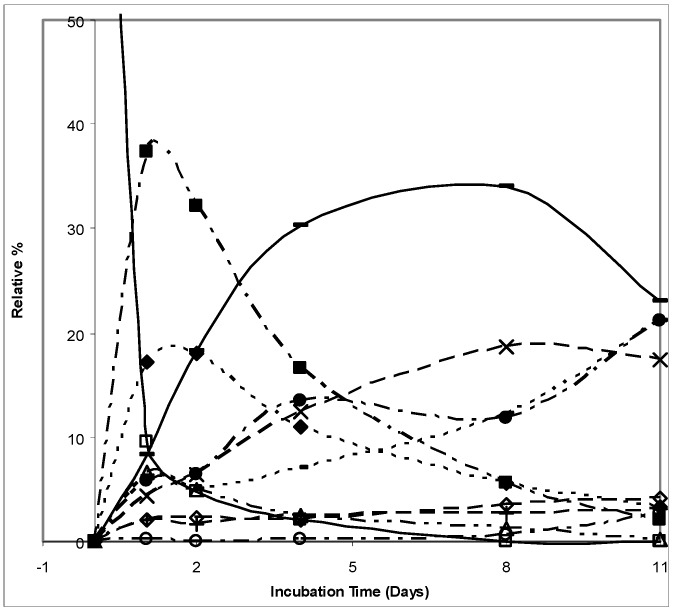
The relative abundance (in %) of (1*S*)-3-carene and its biotransformation products in time-course; Legend: □, (1*S*)-3-carene; ◊, 1,4-cineole; ■, (1*R*)-*p*-menth-1(7),2-dien-8-ol; ∆, (1*R*)-*p*-menth-1,5-dien-8-ol; ▲, (1*S*,3*S*,4*R*)-3,4-epoxycarane; –, *m*-cymen-8-ol; ×, *p*-cymen-8-ol; ○, (1*S*)-2-caranone; ●, (1*S*)-2-caren-4-one; +, (1*S*)-3-caren-2-one; -, (1*S*)-3-caren-5-one.

**Scheme 2:**
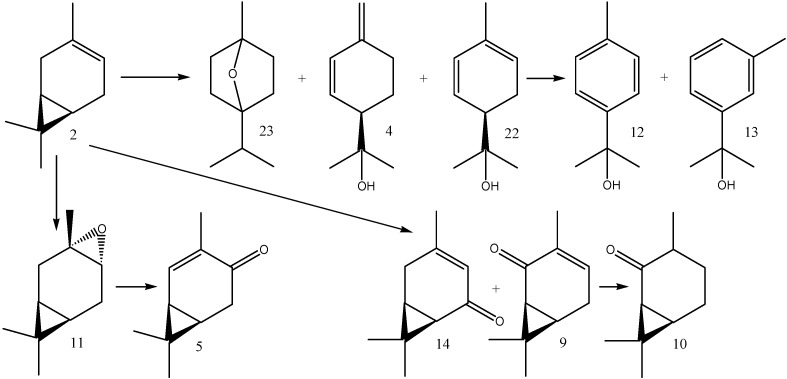
(1*S*)-3-Carene (**2**) and its biotransformation products: 1,4-cineole (**23**) (1*R*)-*p*-mentha-1(7),2-dien-8-ol (**4**), (1*R*)-*p*-mentha-1,5-dien-8-ol (**22**), *p*-cymen-8-ol (**12**), *m*-cymen-8-ol (**13**), (1*S*,3*S*,4*R*)-3,4-epoxycarane (**11**), (1*S*)-2-caren-4-one (**5**), (1*S*)-3-caren-5-one (**14**), (1*S*)-3-caren-2-one (**9**), and (1*S*)-2-caranone (**10**).

Unlike (1*S*)-2-carene (**1**), (1*S*)-3-carene (**2**) was readily transformed with most of the material being consumed within the first day. The most rapidly formed products were (1*S*,3*S*,4*R*)-3,4-epoxycarane (**11**) and (1*R*)-*p*-mentha-1(7),2-dien-8-ol (**4**) whose relative abundances after 24 h were 17% and 37%, respectively. Further on, aromatic products were formed by subsequent dehydrogenation reactions. The relative abundance of *p*-cymen-8-ol (**12**) and *m*-cymen-8-ol (**13**) peaked after eight days at 19% and 34%, respectively (Figure 2). The oxidative transformation of (1*S*,3*S*,4*R*)-3,4-epoxy-carane (**11**) led to the formation of (1*S*)-2-caren-4-one (**5**). Scheme 2 shows the structures of the (1*S*)-3-carene (**2**) biotransformation products.

The quantitative product yields in μg/L are entered in Table 1. The maximum yield was obtained for (1*R*)-*p*-mentha-1(7),2-dien-8-ol (**4**) after one day (58.3 μg/L) and for *m*-cymen-8-ol (**13**) after four days (32.3 μg/L). The total yield of biotransformation products decreased continually with time due to their volatility. The overall yield (3.5%) was the highest the first day and decreased further on, ending with 1% yield after eleven days. The compound recovery from the Sep-Pak cartridges, which was 60%, also affected the yield.

(1*S*)-3-Carene (**2**) has been subjected to many more biotransformation studies than (1*S*)-2-carene (**1**). Several organisms were employed in those studies, leading to distinct biotransformation products. For instance, (1*S*)-3-carene (**2**) was subjected to human liver microsomes whereby (1*S*,3*S*,4*R*)-3,4-epoxycarane (**11**) and (1*S*)-3-carene-10-ol (**15**) were formed [10]. In rabbits, biotransformation of 3-carene (**2**) led to a formation of *m*-cymen-8-ol (**13**), (1*S*,7*S*)-3-caren-9-ol (**16**), (1*S*,7*S*)-3-carene-9-carboxylic acid (**17**) and (1*S*,7*S*)-3-carene-9,10-dicarboxylic acid (**18**) [11].

**Table 1:** The quantitative yields of (1*S*)-3-carene (**2**) biotransformation products.

Compounds (µg/L)/Days	1	2	4	8	11
3-Carene	20.4 ± 1.79	4.9 ± 0.57	2.3 ± 0.15	0	0
1,4-Cineole	4.1 ± 0.28	2.3 ± 0.12	2.3 ± 0.18	2.8 ± 0.25	0
3,4-Epoxycarene	30.9 ± 3.48	19.7 ± 0.92	11.8 ± 1.13	4.7 ± 0.32	1.5 ± 0.19
*p*-Mentha-1(7),2-dien-8-ol	58.3 ± 3.86	36.1 ± 4.31	16.3 ± 1.90	4.9 ± 0.19	0
*p*-Mentha-1,5-dien-8-ol	8.8 ± 0.72	1.1 ± 0.12	0.6 ± 0.15	0	0
*m*-Cymen-8-ol	10.5 ± 0.84	23.3 ± 1.71	32.3 ± 3.18	29.6 ± 1.12	10.9 ± 1.03
*p*-Cymen-8-ol	6.6 ± 0.92	8.3 ± 0.67	13.7 ± 0.95	16.5 ± 1.15	7.9 ± 1.31
3-Carene-4-one	10.9 ± 0.76	7.1 ± 0.08	15.6 ± 1.13	9.6 ± 0.82	9.6 ± 1.69
3-Carene-2-one	4.1 ± 0.25	1.9 ± 0.15	2.8 ± 0.12	2.3 ± 0.38	1.3 ± 0.01
3-Carene-5-one	11.1 ± 0.80	5.8 ± 0.37	7.7 ± 0.57	10.5 ± 1.50	9.6 ± 1.88

**Scheme 3:**
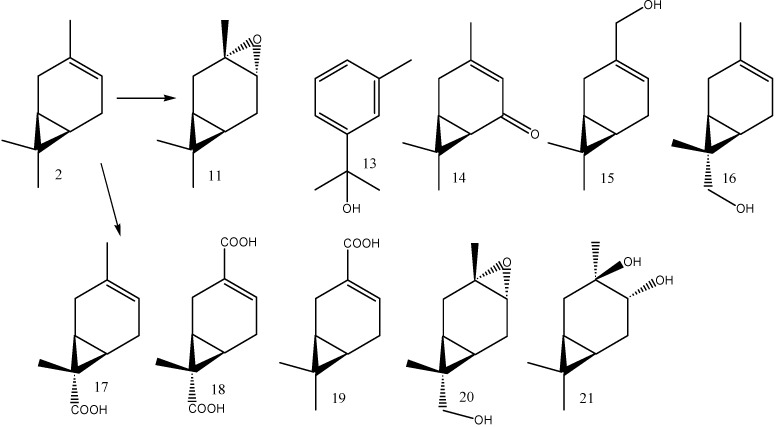
Structures of (1*S*)-3-carene (**2**) biotransformation products formed by various organisms according to literature [8,9,10,11,13].

The bacterium *Acetobacter acetii* transformed (1*S*)-3-carene (**2**) into a mixture of 17 products [12], whereas *Mycobacterium smegmatis* DSM 43061 produced only one major product, (+)-chaminic acid (**19**) [13]. Also, *Spodoptera litura* larvae transformed (1*S*)-3-carene into a single major product, (1*S*,3*S*,4*R*,7S)-3,4-epoxycaran-9-ol (**20**) [9]. In addition, *Nicotiana tabacum* and *Catharanthus roseus* were employed for the biotransformation of (1*S*)-3-carene (**2**) which led to a formation of (1*S*,3*S*,4*R*)-3,4-epoxycarane (**11**), (1*S*,3*R*,4*R*)-3,4-caranediol (**21**), *m*-cymen-8-ol (**13**) and (1*S*)-3-caren-5-one (**14**) [8]. The structures of the mentioned products are given in Scheme 3.

Our carene biotransformation products only partially corresponded to those previously obtained. As expected, they primarily corresponded to those formed by plant tissue cultures [8]. For example, we also identified (1*S*,3*S*,4*R*)-3,4-epoxycarane (**11**) and (1*S*)-3-caren-5-one (**14**) as the products of (1*S*)-3-carene (**2**) biotransformation, however, other compounds, such as (1*R*)-*p*-mentha-1(7),2-dien-8-ol (**4**) and *m*-cymen-8-ol (**13**), were formed in much higher amounts at other time points of the biotransformation time-course.

During our carenes biotransformation, mainly hydroxylated and oxygenated products derived from the limonene (1-methyl-4-(1-methylethenyl)-cyclohexene) and carene structure were formed. The limonene derivatives come from the ring opening of the carene cyclopropane moiety due to the ring strain. Oxidation reactions are common themes in natural product biotransformations owing to the presence of cytochrome P450 (CYP) enzymes.

Some of these products are important scaffolds in the synthesis of more complicated structures. For instance, (1*S*,3*S*,4*R*)-3,4-epoxycarane (**11**) and various hydroxylated limonene derivatives were employed as the starting materials in the synthesis of cannabinoids [14], and (1*S*)-3-caren-5-one (**14**) was utilized in the synthesis of (diphenylphosphinophenyl)pyridine ligands used for the asymmetric synthesis as transition metal complexes [15], and in the synthesis of pyrethroids [16]. (1*S*)-2-Caren-4-one (**5**) was utilized in the total synthesis of chiral oxophosphonates which can be employed in enantioselective catalysis [17]. While some of our products are easily obtained by common organic syntheses [(1*S*,3*S*,4*R*)-3,4-epoxycarane (**11**), (1*R*,4*R*)-*p*-menth-2-en-1,8-diol (**6**)], the synthesis of others is complicated and time-consuming [(1*S*)-2-caren-4-one (**5**), (1*S*)-3-caren-2-one (**9**), (1*S*)-3-caren-5-one (**14**), (1*R*)-*p*-menth-2-en-7,8-diol (**7**)].

The carene biotransformation products also display antifungal and antibacterial properties. *p*-Mentha-1,5-dien-8-ol (**22**) showed significant antimicrobial activity against *Staphylococcus* and *Streptococcus* species [18]. One of the main constituents of *Rhododendron anthopogonoides* essential oil, 1,4-cineole (**23**), exhibited a strong fumigant toxicity against the adults of maize weevil, *Sitophilus zeamais* [19]. Moreover, *Chaerophyllum byzantinum* Boiss. essential oil containing *p*-cymen-8-ol (**12**) as one of the main components exhibited good anticandidal activity against *Candida glabrata* [20].

Importantly, in our case, biotransformation of carenes led to the formation of narrow range of highly abundant compounds which is fundamental for their possible isolation and further utilization. Moreover, the precise selection of biotransformation time-course could maximize the production of the desired compounds. For example, biotransformation of (1*S*)-2-carene (**1**) afforded (1*R*,4*R*)-*p*-menth-2-en-1,8-diol (**6**) and (1*S*)-2-caren-4-one (**5**) as the most abundant products after five and twelve days, respectively, based to their relative abundance. On the other hand, in the biotransformation of (1*S*)-3-carene (**2**), (1*R*)-*p*-mentha-1(7),2-dien-8-ol (**4**) was the most abundant product after 24 h and *m-*cymen-8-ol (**13**) after four and eight days. Unfortunately, the biotransformation has still its main drawback in low overall conversion to products, which in our case was at the most 3.5%.

## 3. Experimental

### 3.1. General

Solvents and reagents. Chloroform-d, *tert*-butylmethylether (99.8%, TBME), (1*S*)-(+)-2-carene (97%), (1*S*)-(+)-3-carene (99%) potassium *tert*-butoxide (97%), 2,2,6,6-tetramethylpiperidine (97%), butyllithium (1.6 M solution in hexane), benzophenone (99%), calcium hydride (95%), sodium (99%), diethylaluminum chloride (1 M solution in hexane), *m*-chloroperbenzoic acid (77%), pyridinium chlorochromate (98%) and eucarvone (2,6,6-trimethylcyclohepta-2,4-dienone) were purchased from Aldrich. Kinetin, 2,4-dichlorophenoxyacetic acid (2,4-D, 98%), 6-benzylaminopurine (BAP) and agar were purchased from Sigma (plant cell culture quality). Sodium sulphate anhydrous (p.a.), sodium bicarbonate (p.a.), hydrochloric acid (37%), pyridine (p.a.), diethyl ether (p.a.), petroleum ether (p.a.), benzene (p.a.), dichloromethane (p.a.), chloroform (p.a.), sodium acetate (p.a.) and sucrose were purchased from Penta Chemicals s.r.o., Czech Republic. The solvents were dried by refluxing with a suitable drying reagent. Sep-Pak C-18 cartridges were purchased from Waters Co. Silica gel (60–200 mesh) for column chromatography, Celite and TLC plates F254 were purchased from Merck Co. The reactions were followed by TLC eluted with petroleum ether-diethylether (1/1 v/v). During column chromatography, the compounds were eluted with petroleum ether-diethylether (2/1–1/1 v/v). The ^1^H- and ^13^C-NMR spectra were recorded on a Bruker AVANCE 500 MHz spectrometer at 499.8 MHz and 125.8 MHz in deuterochloroform using tetramethylsilane (δ 0.0) as internal reference (^1^H-NMR) or central line of residual signal of solvent (δ 77.00 for ^13^C-NMR). ^1^H-NMR data are presented in the following order: chemical shift (δ) expressed in ppm, multiplicity (s, singlet; d, doublet; t, triplet; q, quartet; m, multiplet), coupling constants in Hertz, number of protons. Mass spectra were recorded after separation by gas chromatography (GC: Finnigan Focus GC, injection temperature 200 °C, split/splitless injector) on Fisons MD 800 with EI ionization at 70 eV. MS source temperature was 200 °C. Helium (1 mL/min) was used as a carrier gas. Column: DB-5ms (30 m × 0.25 mm, film thickness 0.25 μm). Temperature program was 50(1)-40-80(15)-4-160(0)-20-320(6). Infrared spectra (IR) were measured after separation by gas chromatography on a Bruker Equinox 55 spectrometer.

### 3.2. Cultivation of in Vitro Cultures

Embryogenic culture of *P. abies* was induced from immature zygotic embryos and maintained on sterile medium, solidified with 0.75% (w/v) agar. The medium was prepared according to Gupta and Durzan [21] and supplemented with 5 μM 2,4-D, 2 μM kinetin, 2 μM BAP and 30 g/L sucrose. Its pH was adjusted to 5.80 ± 0.05 before autoclaving. The cultures were subcultivated every 7 days. The suspension culture was initiated from the embryogenic culture. The same supplemented maintenance medium (excluding agar) was used as the nutrient medium. The suspension cultures were kept on rotary shakers at 100 rpm in 250 mL Erlenmeyer flasks at 24 °C in darkness. The flasks were sealed with aluminium foil.

### 3.3. Autooxidation of Carenes

(1*S*)-2-Carene (**1**) and (1*S*)-3-carene (**2**) (20 μL, 23 mg), respectively, were added to a 100 mL medium without cells in a 250 mL Erlenmeyer flask. The flasks, sealed with aluminium foil, were kept on a rotary shaker at 100 rpm at 24 °C in darkness for one day in case of (1*S*)-3-carene (**2**) and two days for (1*S*)-2-carene (**1**). The experiments were performed in duplicate. After incubation, the medium (50 mL) was applied to a Sep-Pak C-18 syringe cartridge (Waters, Milford). The sugar and salts originating from the nutrient medium were washed out from the cartridge with 10 mL distilled water, whereupon the products were eluted with 1.5 mL of TBME. The resulting samples were analyzed by GC-MS and compared.

### 3.4. Biotransformation of Carenes

(1*S*)-2-Carene (**1**) and (1*S*)-3-carene (**2**) (20 μL, 23 mg), respectively, were added to a 100 mL suspension culture in a 250 mL Erlenmeyer flask, which was sealed with aluminium foil and kept on a rotary shaker at 100 rpm at 24 °C in darkness for up to 12 days. The experiments were performed in duplicate. After incubation, the medium was filtered through a filter paper (No. 388, filtrate volume 50 mL) and then it was applied to a Sep-Pak C-18 syringe cartridge (Waters, Milford). The samples were processed and analysed the same way as autooxidation samples.

### 3.5. Quantification of Product Formation

In case of (1*S*)-3-carene (**2**), the amount of products formed throughout the biotransformation course was quantified. To an eluate from the Sep-Pak cartridge was added 100 μL of 1-adamantol solution of 3 mg/mL concentration as an internal standard. One microlitre of the final solution was subjected to GC-MS.

### 3.6. GC-MS Analyses

The biotransformation products were identified by gas chromatography - mass spectrometry by the use of the MS library Wiley 275 and NIST or by comparisons of their retention times and mass spectra with those of the synthesized reference substances [24,25,26,27,28,29,30]. The relative quantitative yield of each product (in %) was determined as the GC-MS integration area of that product divided by the GC-MS integration area of all products.

### 3.7. Synthesis of Reference Compounds

**Scheme 4:**
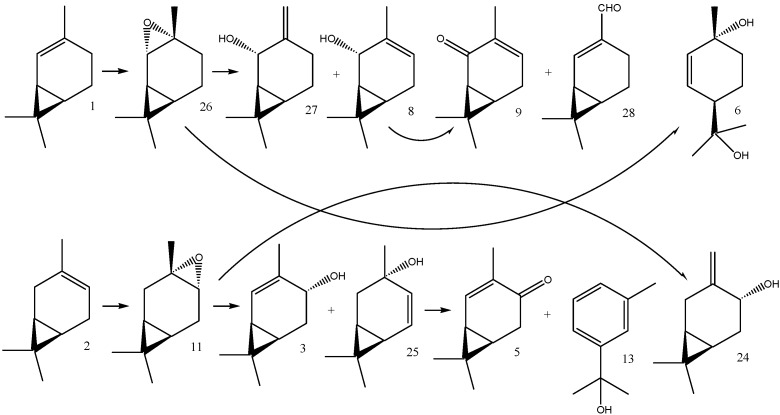
Structures of synthesized reference substances.

These were prepared as shown in Scheme 4.

*(1S,3S,4R)-3,4-Epoxycarane* (**11**) [22]. (1*S*)-3-Carene (2 g, 14.7 mmol) was added to a stirred solution of 3-chloroperbenzoic acid (2.54 g, 14.7 mmol) in dry chloroform (50 mL) and stirred for an additional 3 h. The reaction was quenched with NaHCO_3_ solution, which was then extracted with chloroform. The combined organic solutions were dried and evaporated. The residue (2.2 g, 98.5%) was utilized in the next step without purification. ^1^H-NMR: 0.47 (ddd, *J* = 1.3; 5.2; 5.4 Hz, 1H, H-1), 0.52 (ddd, *J* = 1.4; 5.2; 5.4 Hz, 1H, H-6), 0.76 (s, 3H, H-8), 1.03 (s, 3H, H-9), 1.24 (s, 3H, H-10), 1.54 (dd, *J* = 1.3; 15.7 Hz, 1H, H-2a), 1.66 (dt, *J* = 1.4; 1.4; 15.9 Hz, 1H, H-5a), 2.12 (dd, *J* = 5.4; 15.7 Hz, 1H, H-2b), 2.28 (ddd, *J* = 1.1; 5.2; 15.9 Hz, 1H, H-5b), 2.87 (t, *J* = 1.1 Hz, 1H, H-4). ^13^C-NMR: 13.79 (C-1), 14.56 (C-8), 15.91 (C-6), 15.98 (C-7), 19.13 (C-5), 23.12 (C-10), 23.27 (C-2), 27.68 (C-9), 56.11 (C-3), 58.34 (C-4). MS (EI): 152 (M^+^, 1.3), 137 (41), 123 (17), 119 (17), 109 (75), 95 (32), 91 (23), 81 (50), 67 (94), 55 (34), 43 (100), 41 (55), 39 (33).

*(1S,4R)-3(10)-Caren-4-ol* (**24**) [23]. To a solution of tetramethylpiperidine (4.6 mL, 27 mmol) in dry benzene was added n-butyllithium (10.6 mL, 27 mmol) at 0 °C. After 15 min diethylaluminium chloride (27 mL, 27 mmol) was added. After another 30 min, a solution of (1*S*,3*S*,4*R*)-3,4-epoxycarane (2 g, 13 mmol) in dry benzene was added. The mixture was stirred 30 min and then it was poured into ice-cold 10% hydrochloric acid (50 mL). The mixture was extracted with diethyl ether (3 × 50 mL). The combined organic solutions were dried and evaporated. The residue (1.35 g, 68%) was purified by silica gel column chromatography. ^1^H-NMR: 0.70 (dt, *J* = 3.9; 9.3; 9.3 Hz, 1H, H-6), 0.82 (ddt, *J* = 0.9; 0.9; 8.2; 9.3 Hz, 1H, H-1), 0.88 (s, 3H, H-8), 1.02 (s, 3H, H-9), 1.55 (ddd, *J* = 3.1; 3.9; 15.2 Hz, 1H, H-5a), 2.24 (dddd, *J* = 0.7; 3.4; 9.3; 15.2 Hz, 1H, H-5b), 2.27 (d, *J* = 16.5 Hz, 1H, H-2a), 2.75 (ddt, *J* = 2.6; 2.6; 8.2; 16.5 Hz, 1H, H-2b), 4.10 (t, *J* = 3.2 Hz, 1H, H-4), 4.76 (ddd, *J* = 1.0; 1.9; 2.6 Hz, 1H, H-10a), 4.82 (ddq, *J* = 0.6; 0.6; 0.6; 1.9; 2.6 Hz, 1H, H-10b). ^13^C-NMR: 14.24 (C-8), 15.26 (C-6), 17.52 (C-7), 20.38 (C-1), 24.56 (C-2), 28.61 (C-9), 28.75 (C-5), 71.01 (C-4), 109.05 (C-10), 149.05 (C-3). MS (EI): 152 (M^+^, 3), 137 (16), 134 (25), 119 (32), 109 (44), 95 (43), 92 (84), 91 (87), 83 (55), 81 (45), 79 (42), 77 (26), 69 (38) 67 (46), 55 (100), 53 (32), 43 (40), 41 (87), 39 (43).

*(1S,4R)-2-Caren-4-ol* (**3**) [22]. (1*S*,3*S*,4*R*)-3,4-epoxycarane (2.2 g, 14.5 mmol) was added dropwise at 100 °C to a stirred solution of kalium *terc*-butoxide (2.28 g, 20.7 mmol) in dry pyridine (50 mL). The resulting solution was heated to 135 °C and stirred at this temperature for 3 h. Afterwards, pyridine was removed under reduced pressure and the residue was dissolved in ice-cold water and extracted with petroleum ether (4 × 50 mL). The combined organic solutions were dried and evaporated. The residue (1.6 g, 74%) was a mixture of two products, (1*S*,4*R*)-2-caren-4-ol (**3**) and (1*S*,3*S*)-4-caren-3-ol (**25**) in 2:3 ratio, which had the same R_f_ and thus could not be separated by silica gel column chromatography. **3**: ^1^H-NMR: 1.12 (s, 3H, H-8), 1.21 (s, 3H, H-9), 1.25–1.38 (m, 2H, H-1, H-6), 1.54 (ddd, *J* = 1.8; 5.0; 15.0 Hz, 1H, H-5a), 1.79 (t, *J* = 1.7 Hz, 3H, H-10), 2.05 (ddd, *J* = 3.7; 8.6; 15.0 Hz, 1H, H-5b), 3.72 (dd, *J* = 3.7; 5.0 Hz, 1H, H-4), 5.57 (m, 1H, H-2). ^13^C-NMR: 14.62 (C-8), 17.27 (C-6), 20.77 (C-10), 22.47 (C-7), 22.70 (C-1), 27.80 (C-9), 28.40 (C-5), 67.22 (C-4), 123.11 (C-2), 137.54 (C-3). MS (EI): 152 (M^+^, 1.6), 137 (7), 119 (22), 109 (100), 95 (22), 91 (36), 81 (19), 79 (20), 67 (18), 55 (14), 43 (38), 41 (28), 39 (21). **25**: ^1^H-NMR: 0.86 (s, 3H, H-8), 0.97–1.10 (m, 2H, H-1, H-6), 1.16 (s, 3H, H-9), 1.21 (s, 3H, H-10),1.43 (dd, *J* = 5.2; 15.0 Hz, 1H, H-2a), 2.09 (ddd, *J* = 0.9; 9.3; 15.0 Hz, 1H, H-2b), 5.80 (ddd, *J* = 0.9; 2.5; 9.7 Hz, 1H, H-5), 5.83 (dt, *J* = 0.9; 0.9; 9.7 Hz, 1H, H-4). ^13^C-NMR: 14.66 (C-8), 18.19 (C-9), 22.60 (C-7), 23.81 (C-1), 27.60 (C-10), 28.75 (C-6), 34.44 (C-2), 65.72 (C-3), 128.86 (C-5), 136.43 (C-4). MS (EI): 152 (M^+^, 0.4), 137 (20), 119 (30), 109 (100), 95 (55), 91 (32), 81 (11), 79 (16), 67 (22), 55 (12), 43 (85), 41 (28), 39 (20).

*(1S)-2-Caren-4-one* (**5**) [24]. The mixture of (1*S*,4*R*)-2-caren-4-ol and (1*S*,3*S*)-4-caren-3-ol (0.5 g, 3.3 mmol) was added to a stirred solution of pyridiniumchlorochromate (1.42 g, 6.6 mmol) and sodium acetate (92 mg, 1.1 mmol) in dry dichloromethane. The resulting solution was stirred at RT for 4 h. Then, the solution was poured into diethyl ether (50 mL). The solids formed were filtered off through celite and the filtrate was evaporated under reduced pressure. The residue (0.4 g, 80%) was a mixture of two compounds, (1*S*)-2-caren-4-one (**5**) and *m*-cymen-8-ol (**13**) in 2:3 ratio, which were measured by GC-MS and GC-IR. Then, it was purified by silica gel column chromatography which afforded only *m*-cymen-8-ol as the final product (2-caren-4-one decomposed on column). **5**: MS (EI): 150 (M^+^, 22), 135 (10), 122 (5), 108 (75), 107 (100), 91 (60), 79 (52), 77 (34), 65 (14), 53 (20), 43 (18), 41 (45), 39 (59). IR: 3009, 2966, 2933, 2899, 2839, 1678, 1575, 1463, 1451, 1428, 1371, 1330, 1241, 1142, 1051, 1003, 906, 843. **13** (0.23 g): ^1^H-NMR: 1.58 (s, 6H, H-8, H-9), 2.37 (s, 3H, H-10), 7.06 (d, *J* = 7.8 Hz, 1H, H-4), 7.24 (t, *J* = 7.8 Hz, 1H, H-5), 7.28 (dt, *J* = 1.7; 1.7; 7.8 Hz, 1H, H-6), 7.32 (t, *J* = 1.7 Hz, 1H, H-2). ^13^C-NMR: 21.60 (C-10), 31.75 (C-8, C-9), 72.50 (C-7), 121.37 (C-2), 125.11 (C-6), 127.41 (C-4), 128.13 (C-5), 137.80 (C-3), 149.07 (C-1). MS (EI): 150 (M^+^, 11), 135 (62), 91 (21), 77 (5), 65 (10), 43 (100).

*(1S,2S,3R)-2,3-Epoxycarane* (**26**) [25]. (1*S*)-2-Carene (2 g, 14.7 mmol) was added to a stirred solution of 3-chloroperbenzoic acid (2.54 g, 14.7 mmol) in dry diethyl ether (40 mL) at 0 °C. The resulting solution was stirred overnight. The reaction was quenched with NaHCO_3_ solution which was then extracted with diethyl ether. The combined organic solutions were dried and evaporated. The residue (2.0 g, 90%) was utilized in the next step without purification. ^1^H-NMR: 0.66 (dddd, *J* = 0.8; 2.9; 6.2; 9.0 Hz, 1H, H-6), 1.06 (s, 3H, H-8), 1.07 (dd, *J* = 2.1; 9.0 Hz, 1H, H-1), 1.08 (s, 3H, H-9), 1.26 (s, 3H, H-10), 1.54 (dddt, *J* = 0.5; 2.9; 5.9; 5.9; 14.3 Hz, 1H, H-5a), 1.65–1.70 (m, 2H, H-4), 1.90 (dddd, *J* = 6.7; 7.6; 8.6; 14.3 Hz, 1H, H-5b), 3.02 (d, *J* = 2.1 Hz, 1H, H-2). ^13^C-NMR: 16.45 (C-9), 16.59 (C-5), 20.77 (C-7), 21.07 (C-6), 21.93 (C-10), 23.80 (C-8), 27.14 (C-4), 29.00 (C-1), 57.97 (C-2), 58.27 (C-3). MS (EI): 152 (M^+^, 0.5), 137 (27), 124 (67), 109 (94), 95 (45), 91 (21), 83 (34), 81 (57), 79 (37), 77 (22), 69 (26), 67 (80), 55 (53), 53 (30), 43 (100), 41 (81), 39 (54).

*(1*R*,4*R*)-*p*-menth-2-ene-1,8-diol* (**6**) [26]. To a solution of (1*S*,2*S*,3*R*)-2,3-epoxycarane (0.6 g, 4.4 mmol) in ethyl acetate (15 mL) at 0 °C was added dropwise a solution of acetic acid (125 μL) in water (1 mL). The reaction was stirred for 6 h and then it was concentrated under reduced pressure. The residue was purified by silica gel column chromatography to give 0.5 g (67%) of pure material. ^1^H-NMR: 1.16 (s, 3H, H-10), 1.21 (d, *J* = 0.3 Hz, 3H, H-8), 1.28 (d, *J* = 0.6 Hz, 3H, H-9), 1.40 (ddt, *J* = 2.9; 10.3; 10.3; 13.3 Hz, 1H, H-5a), 1.65 (dddd, *J* = 0.6; 3.1; 12.3; 13.3 Hz, 1H, H-6a), 1.86 (m, 1H, H-5b), 1.93 (dddd, *J* = 1.4; 2.8; 5.0; 12.4 Hz, 1H, H-6b), 2.16 (ddt, *J* = 1.8; 1.8; 5.5; 13.3 Hz, 1H, H-4), 5.73 (ddt, *J* = 1.5; 1.5; 10.3; 15.0 Hz, 2H, H-2, H-3). ^13^C-NMR: 22.94 (C-5), 26.12 (C-10), 27.85 (C-8), 28.23 (C-9), 38.27 (C-6), 46.94 (C-4), 69.45 (C-1), 72.61 (C-7), 127.79 (C-3), 136.56 (C-2). MS (EI): 152 (M^+^-H_2_O, 1), 137 (10), 119 (8), 109 (7), 94 (99), 91 (17), 79 (82), 77 (15), 59 (100), 43 (44), 41 (16), 39 (10).

*(1S,2S)-3-Caren-2-ol* (**8**) [22]. The method was identical to that described for the preparation of (1*S*,4*R*)-2-caren-4-ol. (1*S*,2*S*,3*R*)-2,3-Epoxycarane (2.2 g, 14.5 mmol) was used as the starting material. The residue (1.7 g, 75%) was a mixture of two compounds (1*S*,2*S*)-3-caren-2-ol (**8**) and (1*S*,2*S*)-3(10)-caren-2-ol (**27**) which were purified by silica gel column chromatography. **8** (0.8 g): ^1^H-NMR: 0.76 (ddt, *J* = 0.9; 0.9; 7.8; 8.7 Hz, 1H, H-6), 0.84 (s, 3H, H-9), 0.89 (d, *J* = 8.7 Hz, 1H, H-1), 1.05 (s, 3H, H-8), 1.73 (dt, *J* = 2.0; 2.2; 2.2 Hz, 3H, H-10), 2.05 (dt, *J* = 3.6; 3.6; 19.5 Hz, 1H, H-5a), 2.46 (ddddq *J* = 1.7; 2.0; 2.0; 2.0; 3.5; 7.8; 19.5 Hz, 1H, H-5b), 3.89 (s, 1H, H-2), 5.36 (ddtq, *J* = 0.9; 0.9; 1.8; 1.8; 1.8; 3.4; 4.2 Hz, 1H, H-4). ^13^C-NMR: 13.32 (C-8), 16.91 (C-6), 20.85 (C-10), 21.40 (C-5), 27.60 (C-1), 28.51 (C-9), 65.68 (C-2), 123.62 (C-4), 133.39 (C-3). MS (EI): 152 (M^+^, 14), 137 (25), 134 (9), 119 (25), 109 (92), 95 (49), 94 (54), 91 (50), 82 (72), 81 (57), 79 (40), 77 (33), 69 (51) 67 (67), 59 (26), 55 (44), 53 (32), 43 (54), 41 (100), 39 (97). **27**: (0.8 g): ^1^H-NMR: 0.80 (dd, *J* = 1.3; 8.7 Hz, 1H, H-1), 0.86 (ddd, *J* = 3.7; 8.7; 9.2 Hz, 1H, H-6), 0.92 (s, 3H, H-9), 1.02 (s, 3H, H-8), 1.35 (dddd, *J* = 3.7; 5.2; 13.0; 14.2 Hz, 1H, H-5a), 1.93 (ddd, *J* = 2.5; 5.2; 13.0 Hz, 1H, H-4a), 2.10 (dddd, *J* = 2.5; 6.6; 9.2; 14.2 Hz, 1H, H-5b), 2.29 (ddt, *J* = 1.7; 1.7; 6.6; 13.0 Hz, 1H, H-4b), 4.28 (s, 1H, H-2), 4.83 (dd, *J* = 1.8; 2.0 Hz, 1H, H-10a), 4.94 (dd, *J* = 1.7; 1.8 Hz, 1H, H-10b). ^13^C-NMR: 14.79 (C-9), 19.38 (C-6), 20.62 (C-5), 27.53 (C-4), 28.98 (C-8), 29.10 (C-1), 69.59 (C-2), 111.50 (C-10), 151.11 (C-3). MS (EI): 152 (M^+^, 2.2), 137 (15), 119 (7), 109 (100), 95 (20), 91 (19), 81 (29), 79 (21), 69 (39) 67 (28), 55 (28), 53 (15), 41 (65), 39 (51).

*(1S)-3-Caren-2-one* (**9**) [23,27,28]. The method was identical to that described for the preparation of (1*S*)-2-caren-4-one. (1*S*,2*S*)-3-caren-2-ol (0.5 g, 3.3 mmol) was used as the starting material. The residue (0.35 g, 70%) was a mixture of two compounds (1*S*)-3-caren-2-one (**9**) and (1*S*)-2-caren-10-al (**28**) which were purified by silica gel column chromatography. **9**: (0.1 g): ^1^H-NMR: 1.08 (s, 3H, H-8), 1.18 (s, 3H, H-9), 1.43 (tt, *J* = 1.2; 8.0 Hz, 1H, H-6), 1.64 (dd, *J* = 1.7; 7.8 Hz, 1H, H-1), 1.76 (dt, *J* = 1.6; 1.6; 2.5 Hz, 3H, H-10), 2.44 (m, 1H, H-5a), 2.70 (dddq, *J* = 2.5; 2.5; 2.5; 3.6; 8.3; 21.4 Hz, 1H, H-5b), 6.40 (m, 1H, H-4). ^13^C-NMR: 14.27 (C-8), 16.09 (C-10), 21.87 (C-7), 23.02 (C-5), 26.33 (C-6), 28.48 (C-9), 34.33 (C-1), 135.01 (C-3), 142.57 (C-4), 196.58 (C-2). MS (EI): 150 (M^+^, 100), 135 (52), 109 (23), 108 (57), 107 (86), 95 (14), 91 (62), 79 (44), 77 (25), 67 (43), 65 (14), 55 (12), 53 (22), 51 (13), 43 (14), 41 (37), 39 (40). IR: 3336, 3013, 2965, 2937, 2892, 2841, 2747, 2666, 1678, 1659, 1455, 1434, 1377, 1333, 1241, 1138, 1048, 998, 903, 844. **28**: (0.2 g): ^1^H-NMR: 0.92 (s, 3H, H-8), 1.22 (s, 3H, H-9), 1.33 (m, 1H, H-6), 1.43 (dd, *J* = 5.6; 7.6 Hz, 1H, H-1), 1.70–1.75 (m, 1H, H-5a), 1.82–1.86 (m, 1H, H-4a), 1.92–1.97 (m, 1H, H-5b), 2.41 (m, 1H, H-4b), 7.06 (ddt, *J* = 1.3; 1.3; 3.0; 5.6 Hz, 1H, H-2), 9.37 (s, 1H, H-10). ^13^C-NMR: 15.94 (C-9), 16.22 (C-5), 18.77 (C-4), 25.10 (C-1), 28.39 (C-6), 29.44 (C-8), 31.36 (C-7), 138.08 (C-3), 152.27 (C-2), 193.80 (C-10). MS (EI): 150 (M^+^, 41), 135 (22), 121 (77), 107 (100), 105 (47), 93 (36), 91 (67), 79 (100), 77 (67), 67 (13), 65 (15), 53 (17), 51 (18), 43 (35), 41 (36), 39 (31). IR: 3384, 3010, 2941, 2879, 2803, 2711, 1704, 1638, 1458, 1397, 1298, 1167, 1058, 981, 876, 824, 701.

## 4. Conclusions

Biotransformation of (1*S*)-2-carene and (1*S*)-3-carene leads to the formation of an interesting range of products which are useful precursors of more complicated compounds. Predominantly, hydroxylated and oxygenated compounds are formed. By the proper choice of biotransformation duration, the required compounds may be generated selectively which may simplify their isolation, although the overall yield of the biotransformations is rather low yet comparable to that seen in similar biotransformations.
